# Efficacy and safety of immune checkpoint inhibitors combined with chemotherapy in patients with extensive-stage small cell lung cancer: a systematic review and meta-analysis of randomized controlled trials

**DOI:** 10.3389/fonc.2023.1151769

**Published:** 2023-04-19

**Authors:** Chunlan Chen, Peng Tian, Jiangshan Zhong, Xianming Fan

**Affiliations:** Department of Respiratory and Critical Care Medicine, The Affiliated Hospital of Southwest Medical University, Luzhou, China

**Keywords:** small cell lung cancer, chemotherapy, immune checkpoint inhibitors, randomized controlled trial, meta-analysis

## Abstract

**Objective:**

Many clinical trials of immune checkpoint inhibitors (ICIs) in combination with chemotherapy in the first-line treatment of extensive-stage small cell lung cancer (ES-SCLC) have been initiated, but the conclusions of these trials are not identical. This meta-analysis aimed to comprehensively collect these randomized clinical controlled trials (RCTs) to evaluate the efficacy and safety of ICIs combined with chemotherapy in the first-line treatment of ES-SCLC.

**Methods:**

We systematically searched PubMed, Embase, and ClinicalTrials databases, to find relevant studies published until October 2022.RevMan 5.4 software was used for statistical analysis. The Cochrane Risk of Bias Tool was adopted to evaluate the risk of bias in the included studies. The primary outcome of this study was overall survival (OS), while the secondary outcomes were progression-free survival (PFS), objective response rate (ORR), all grand AEs (AEs), and ≥ 3 grand adverse events (≥ 3 AEs).

**Results:**

A total of 780 articles were obtained in the initial examination, which was screened by layer and finally included 8 studies including 3367 patients. Six studies evaluated the efficacy of PD-1/PD-L1 inhibitors (Pembrolizumab, Nivolumab, Atezolizumab, Durvalumab, Adebrelimab, Serpulimab) combined with chemotherapy, and two studies evaluated the efficacy of CTLA-4 inhibitors (Ipilimumab) in combination with chemotherapy. The results showed that compared to chemotherapy alone, ICIs combined with chemotherapy significantly improved patients’ OS (HR=0.8, 95% CI (0.72-0.85), P<0.05), PFS (HR = 0.72, 95% CI (0.63-0.83), P < 0.05), and ORR(RR=1.08, 95% CI: 1.03-1.13, P<0.05), but patients would experience more any grand AEs and ≥3 grand AEs. Subgroup analysis showed that the PD-1/PD-L1 group performed better than the CTLA-4 group in both efficacy and safety. And ICIs plus chemotherapy significantly improved OS and PFS in patients regardless of age, gender, and performance status.

**Conclusion:**

The addition of ICIs to chemotherapy resulted in significant improvements in both PFS and OS for patients with ES-SCLC, but patients would experience more AEs.

## 1 Introduction

Globally, lung cancer is the second most common cancer and one of the main causes of death due to cancer. In 2020, 1.8 million people died from lung cancer (1). Small cell lung cancer (SCLC) accounts for about 15% of lung cancer, characterized by high aggressiveness and lethality. Due to its rapid progression and early onset of metastasis, about 60-70% of SCLC patients are diagnosed with extensive small cell lung cancer (ES-SCLC) when found diseased (2). SCLC is highly sensitive to chemotherapy, so chemotherapy usually shows obvious treatment effects in the early stage, and the remission rate can reach 60% ~ 80%. However, most patients will progress or relapse in a short time after early chemotherapy, and the relapsed tumor is resistant to further treatment (3, 4). In the past 40 years, etoposide or irinotecan and platinum remain the standard first-line treatments for ES-SCLC. But even with standard first-line chemotherapy, the median overall survival (OS) of patients is only about 10 months (5, 6), the average OS is only 2 ~ 4 months (7, 8), and the 5-year survival rate is only about 7% (9). Therefore, finding better treatment options for the majority of patients has become a new goal for researchers.

In recent years, the advent of immunotherapy, especially immune checkpoint inhibitors (ICIS), has changed the traditional treatment paradigm for multiple tumors. Tumor cells can escape from attacks by the immune system through multiple mechanisms, and their cell surface-expressed immunosuppressive molecules can secrete immunosuppressive factors and can also recruit other suppressive immune cell groups (10). Specific inhibitors against checkpoint receptors can block this immunosuppression, thereby increasing the specific immune response of T lymphocytes and eliciting an antitumor response (11, 12). Currently, the most common ICIS used in clinical practice is monoclonal antibodies (mAbs) against programmed death receptor 1 (PD-1) and its ligand 1 (PD-L1), cytotoxic T-lymphocyte antigen 4 (CTLA-4).ICIs have achieved phased success in the treatment of non-small cell lung cancer, melanoma, gastric cancer, liver cancer, bladder cancer, kidney cancer, and other malignant tumors in the past decade (13).

Based on the results of the checkmate-032 (14) study, nivolumab was approved by the US Food and Drug Administration (FDA) in 2018 for the third-line treatment of ES-SCLC. The phase III randomized trial IMpower-133, CASPIAN demonstrated the addition of Atezolizumab or Durvalumab to first-line chemotherapy can improve progression-free survival (PFS) and OS, respectively, with results still favoring the combination arm after three years of follow-up. Therefore, the FDA subsequently approved Atezolizumab or Durvalumab combined with chemotherapy for the first-line treatment of ES-SCLC (15). But not all researches show the benefit of ICIs for ES-SCLC patients. In KEYNOTE-604 (16), the improved OS of patients treated with Pembrolizumab plus chemotherapy compared with chemotherapy alone was not evident. The same was true for the combination of Nivolumab and chemotherapy in another phase II study, EA5161, in which the median PFS was 5.5 months and 4.7 months for Nivolumab plus chemotherapy and chemotherapy alone, respectively.

Therefore, this study aims to collect data from these trials more comprehensively around the world, evaluate the efficacy and safety of ICIs for ES-SCLC, and provide some reference for the clinical selection of treatment for small cell lung cancer.

## 2 Methods

### 2.1 Search strategy

According to the Preferred Reporting Items for Systematic Reviews and Meta-analyses (PRISMA) reporting guidelines (17, 18), we systematically searched PubMed, Embase, and ClinicalTrials databases, to find relevant studies published until October 2022. The main search terms and combinations include extensive staging, SCLC, ICIs, and randomized controlled trials. The manual search of the list of references for all available reviews was also conducted to confirm the final selection. Three reviewers independently conducted literature searches. Three researchers conducted the literature search independently.

### 2.2 Inclusion and exclusion criteria

Establish inclusion and exclusion criteria based on the principles of PICOS (Population, Intervention, Comparison, Output, Study design).

Inclusion criteria: (1) randomized controlled trial (RCT); (2) Patients with pathologically confirmed ES-SCLC who have not previously received treatment, regardless of race, age, or gender; (3) Intervention measures: ICIs plus chemotherapy versus placebo combined with chemotherapy or chemotherapy alone; (4) Outcome measures: overall survival (OS), progression-free survival (PFS), objective response rate (ORR), and ≥ 3 grand adverse events (AEs).

Exclusion criteria: (1) Non-randomized controlled trials or retrospective studies; (2) Unable to obtain outcome measures indicators or full text; (3) Repeated publications; (4) Non-Chinese and English literature.

### 2.3 Data extraction

Two researchers independently screen the literature and extract data. If there are differences, they will negotiate with a third researcher to resolve them. Data extraction included: the author, year of publication, study design, number of patients, specific intervention plans, and related outcome measures. The Cochrane Risk of Bias Tool was adopted to evaluate the risk of bias in the included studies (19).

### 2.4 Statistical analysis

RevMan 5.4 software was used for statistical analysis. Hazard ratio (HR) for survival outcomes (OS and PFS), odds ratio (OR) for dichotomous outcomes (ORR and AEs), and their 95% confidence interval (CI) were used to measure outcomes and safety. Heterogeneity between included studies was quantified by combining I2 to determine the magnitude of heterogeneity. If the heterogeneity across the studies is small, a fixed effect model was used for meta-analysis; If there is significant heterogeneity across studies, the source of heterogeneity was further analyzed, and a random effects model was used for meta-analysis after excluding the effect of obvious clinical heterogeneity. P < 0.05 was taken as statistically significant.

## 3 Results

### 3.1 Literature search

**Figure 1:**
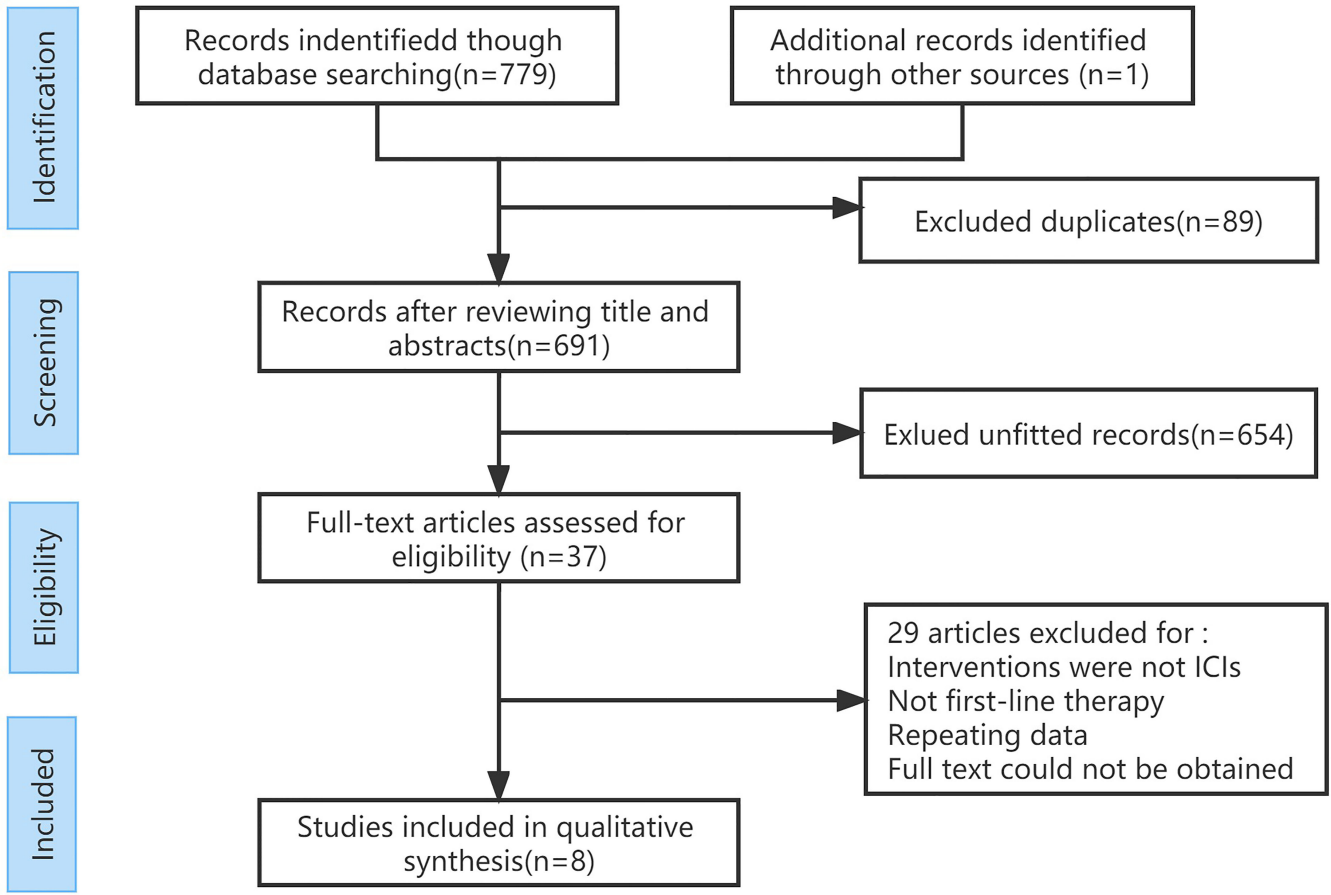
PRISMA Flowchart of the identification of eligible studies.

**Figure 2:**
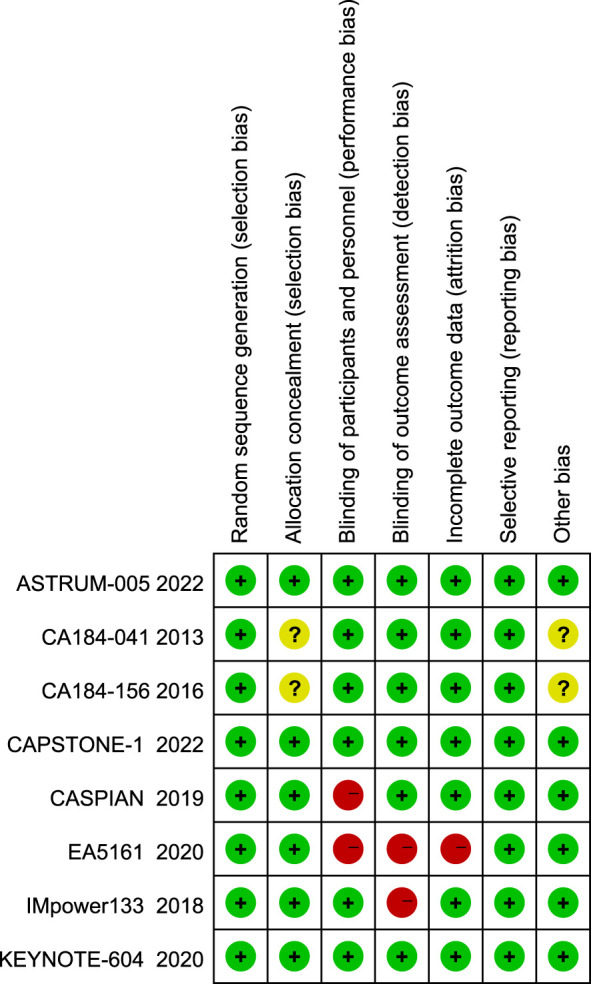
Methodological quality and risk of included trials.

A total of 780 articles were obtained in the initial examination, which was screened by layer and finally included 8 studies including 3367 patients. The literature screening flow is shown in **Figure 1**. **Table 1** summarizes the basic characteristics of all included studies. We adopted the Cochrane risk of bias tool to evaluate the quality of the included studies, and the results of the studies are shown in **Figure 2**.

**Table 1:** Characteristics of studies included in the meta-analysis.

Author	Publication Year	Study Name	NCT	Phase	Intervention arm	No. of patients	Control arm	No. of patients	Tumor assessment criteria	OS	PFS	ORR(%)	≥3AEs		all AEs	
										HR (95% CI)	HR (95% CI)		inervention	control	inervention	control
Reck	2013	CA184-041	NCT00527735	II	Ipi+PC	42	PC	45	mWHO/irRC	0.75(0.46-1.23)	0.93(0.59-1.45)	57 vs 49	17/42	13/44	36/42	40/44
Reck	2016	CA184-156	NCT01450761	III	Ipi+EP	478	EP	476	mWHO	0.94(0.81-1.09)	0.85(0.75-0.97)	62 vs 62	231/478	214/476	391/478	361/476
Horn Reck	2018	IMpower133	NCT02763579	III	Ate+CE	201	Pla+CE	202	RECIST V.1.1	0.76(0.60-0.95)	0.77(0.62-0.96)	60.2 vs 64.4	115/198	113/196	188/198	181/196
Paz-ares Luis	2019	CASPIAN	NCT03043872	III	Dur+EP/CE	268	EP/CE	269	RECIST V.1.1	0.71(0.6-0.86)	0.80(0.66-0.96)	67.9 vs 58.0	163/265	166/266	260/265	258/266
Rudin	2020	KEYNOTE-604	NCT03066778	III	Pem+EP/CE	228	Pla+EP/CE	225	RECIST V.1.1	0.78(0.63-0.97)	0.75(0.61-0.91)	70.6 vs 61.8	171/223	167/222	223/223	222/223
leal T	2020	EA5161	NCT03382561	II	Niv+EP/CE	80	EP/CE	80	RECIST V.1.1	0.67(0.46-0.98)	0.65(0.46-0.91)	52.3 vs 47.7	62/80	48/80	NR	NR
Wangjie	2022	CAPSTONE-1	NCT03711305	III	Ade+CE	230	pla+CE	232	RECIST V.1.1	0.72(0.58-0.90)	0.67(0.54-0.83)	70.4 vs 65.9	197/230	197/232	230/230	230/232
YingCheng	2022	ASTRUM-005	NCT04063163	III	Ser+CE	389	CE	196	RECIST V.1.1	0.63(0.49-0.82)	0.48(0.38-0.59)	80.2 vs 70.4	129/389	54/196	372/389	191/196

Ipi, Ipilimumab; Ate, Atezolizumab; Dur, Durvalumab; Pem, Pembrolizumab; Niv, Nivolumab; Ade, Adebrelimab; Ser, Serplulimab;

PC, paclitaxel+cisplatin; EP, etoposide+cisplatin; EC, etoposide+carboplatin; Pla, placebo.

Six studies evaluated the efficacy of PD-1/PD-L1 inhibitors (Pembrolizumab, Nivolumab, Atezolizumab, Durvalumab, Adebrelimab, Serpulimab) combined with chemotherapy, and two studies evaluated the efficacy of CTLA-4 inhibitors (Ipilimumab) in combination with chemotherapy. All studies reported on OS, PFS, ORR, and AEs, and we also performed subgroup analyses by gender, age, and performance status subgroups based on information published by the studies.

### 3.2 Results of meta-analysis

The primary outcome of this study was OS, while the secondary outcomes were PFS, ORR, and AEs.

#### 3.2.1 OS

**Figure 3:**
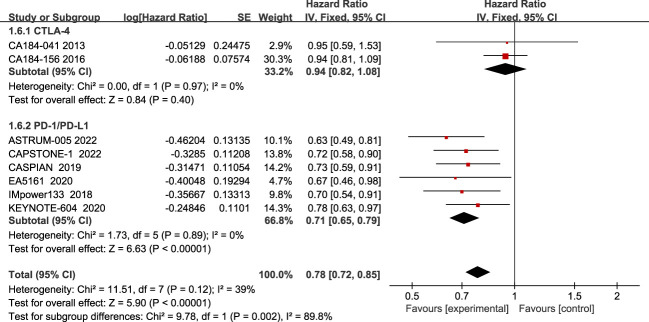
Forest plot of OS comparison between ICIs combined with chemotherapy and chemotherapy alone and subgroup analysis.

Regarding OS, eight studies have reported OS, with no significant heterogeneity among all studies (I2 = 39%; **Figure 3**). The results showed that compared to chemotherapy alone, ICIs combined with chemotherapy significantly improved patients’ OS (HR=0.78, 95% CI:0.72-0.85, P<0.05).

The results of the subgroup analysis showed that PD-1/PD-L1 inhibitors combined with chemotherapy significantly improved OS in patients(HR=0.71, 95% CI:0.65-0.79, P<0.05), while there was no statistically significant improvement in the CTLA-4 inhibitor group(HR=0.94, 95% CI:0.82-1.08, P=0.40).

**Figure 4:**
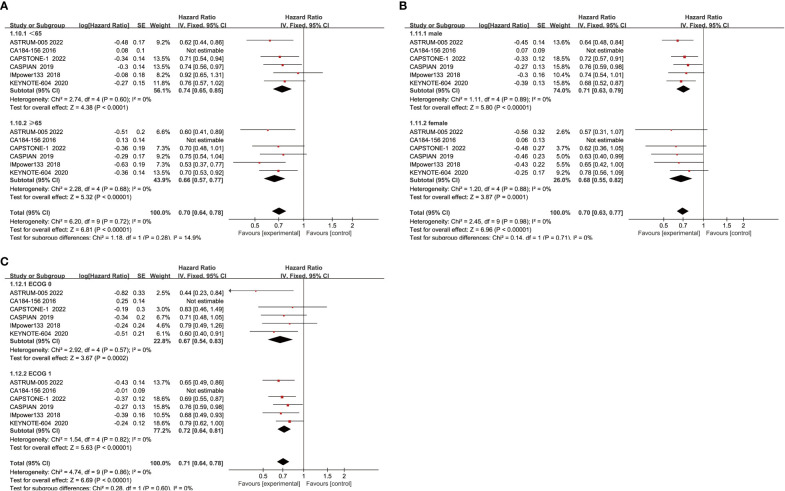
**(A)** OS-age; **(B)** OS-sex; **(C)** OS- performance status.

When subgroup analysis was based on age, gender, and performance status, the results showed that ICIs combined with chemotherapy significantly improved OS in patients compared with chemotherapy alone, regardless of whether they were male(HR=0.71, 95% CI:0.63-0.79, P<0.05) or female(HR=0.68, 95% CI:0.55-0.82, P<0.05), aged ≥65 years(HR=0.66, 95% CI:0.57-0.77, P<0.05) or <65 years(HR=0.74, 95% CI:0.65-0.85, P<0.05), and performance status score of 0(HR=0.67, 95% CI:0.54-0.83, P<0.05) or 1(HR=0.72, 95% CI:0.64-0.81, P<0.05) (**Figure 4**).

#### 3.2.2 PFS

**Figure 5:**
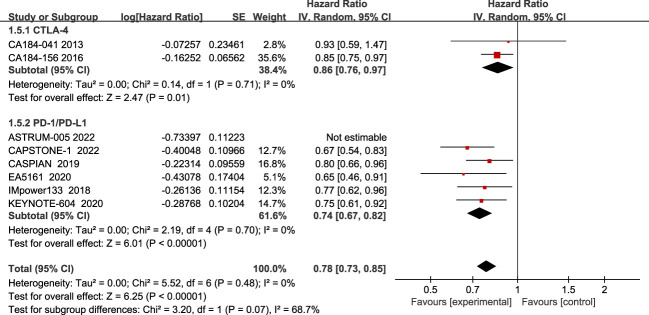
Forest plot of PFS comparison between ICIs combined with chemotherapy and chemotherapy alone and subgroup analysis.

Regarding PFS, all eight included studies reported PFS outcome measures, and a random effects model was used because of heterogeneity among studies (I2 = 69%; **Figure 5**). The results showed that the treatment regimen of ICIS combined with chemotherapy significantly improved PFS in patients, and the difference was statistically significant (HR = 0.72, 95% CI:0.63-0.83, P < 0.05).

Subgroup analysis found a large heterogeneity among PD-1/PD-L1 inhibitor groups, and after excluding the study ASTRUM-005 using a case-by-case exclusion method, there was no heterogeneity (I2 = 0%), and the results showed that PD-1/PD-L1 inhibitors significantly improved PFS in patients(HR=0.74, 95% CI:0.67-0.82, P<0.05).In contrast, there was no heterogeneity between studies in the CTLA-4 group, indicating that its combination with chemotherapy improves patients’ PFS compared to chemotherapy alone(HR=0.86, 95% CI:0.76-0.97, P<0.05).

**Figure 6:**
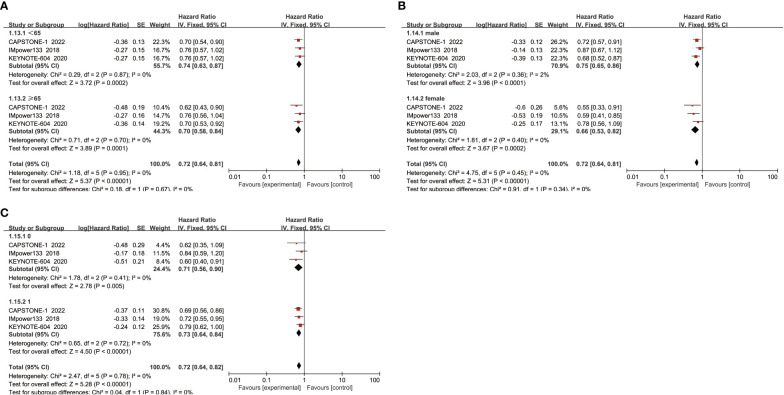
**(A)** PFS-age; **(B)** PFS-sex; **(C)** PFS- performance status.

When subgroup analysis was conducted for age, gender, and performance status, it was found that regardless of age<65 years(HR=0.74, 95% CI:0.63-0.87, P<0.05) or ≥ 65 years(HR=0.70, 95% CI:0.58-0.84, P<0.05), male(HR=0.75, 95% CI:0.65-0.86, P<0.05) or female(HR=0.66, 95% CI:0.53-0.82, P<0.05), and ECOG score was 1(HR=0.73, 95% CI:0.64-0.84, P<0.05) or 0(HR=0.71, 95% CI:0.56-0.90, P<0.05), ICIs combined with chemotherapy could effectively improve the PFS of patients, with a statistically significant difference (**Figure 6**).

#### 3.2.3 ORR

**Figure 7:**
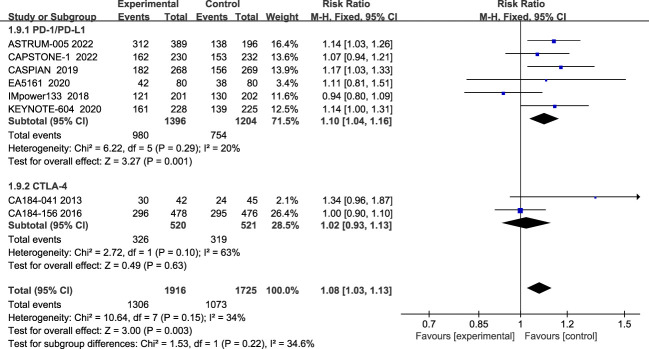
Forest plot of ORR comparison between ICIs combined with chemotherapy and chemotherapy alone and subgroup analysis.

Regarding ORR, eight studies have reported ORR, and the heterogeneity among the studies is not significant (I^2 =^ 34%; **Figure 7**). Compared with the chemotherapy group, the ORR of the combined treatment group was significantly improved (68.2% vs 62.2%), (RR=1.08, 95% CI: 1.03-1.13, P<0.05). Subgroup analysis showed that patients in the PD-1/PD-L1 inhibitor group had significantly improved ORR (RR=1.10, 95% CI:1.04-1.16, P<0.05), while the CTLA-4 inhibitor group did not show a significant difference in improving ORR compared to the chemotherapy alone (RR=1.02, 95% CI: 0.93-1.13, P=0.63).

#### 3.2.4 Safety analysis

**Figure 8:**
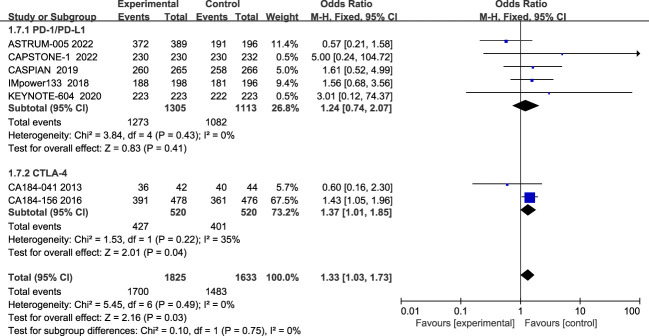
Forest plot of all grade AEs comparison between ICIs combined with chemotherapy and chemotherapy alone and subgroup analysis.

Seven studies reported all treatment-related adverse events, and heterogeneity among studies was not evident(I2 = 0%; **Figure 8**). The most common of all grade AEs were neutropenia, anemia, leukopenia, thrombocytopenia, nausea, diarrhea, alopecia, etc. The results showed that patients treated with ICIs plus chemotherapy had more all-grade treatment-related adverse events than those treated with chemotherapy alone (HR=1.33, 95% CI:1.03-1.73, P<0.05). Subgroup analysis showed that patients in the CTLA-4 inhibitor group (HR=1.37, 95% CI:1.01-1.85, P<0.05) and patients treated with PD-1/PD-L1 inhibitors (HR=1.24, 95% CI:0.74-2.07, P=0.41) all had more all-grade treatment-related adverse events than chemotherapy alone, although the difference was not statistically significant.

**Figure 9:**
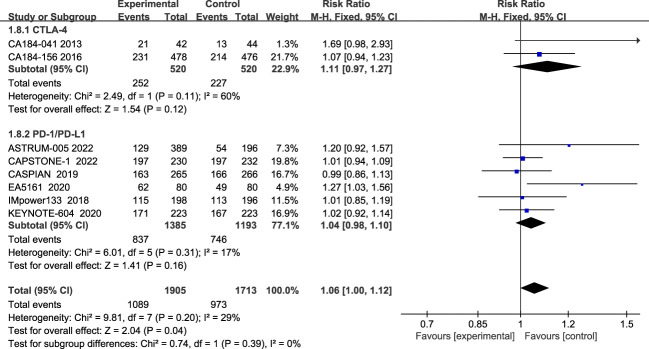
Forest plot of grade≥3AEs comparison between ICIs combined with chemotherapy and chemotherapy alone and subgroup analysis.

The incidence of grade 3 and above AES during treatment was reported in eight studies, with insignificant heterogeneity among studies (I2 = 29%; **Figure 9**). The most common grade 3 and above AEs were mainly in the hematologic, such as neutropenia, anemia, thrombocytopenia, leucopenia, etc., while the main manifestations outside the hematological system are nausea, alopecia, and impaired liver function, etc. According to the results, patients treated with ICIs plus chemotherapy had more grade 3 and higher chemotherapy-related adverse events than patients treated with chemotherapy alone (RR=1.06; 95% CI:1.00-1.12; P=0.04). Subgroup analysis showed that patients in the CTLA-4 inhibitor group (RR=1.11; 95% CI (0.97-1.27); P=0.12) and patients in the PD-1/PD-L1 inhibitor group (RR=1.04; 95% CI, 0.98-1.10; P=0.16) both had more grade 3 and higher adverse events than chemotherapy alone, although the differences were not statistically significant.

### 3.3 Heterogeneity and publication bias

In the present study, the only significantly heterogeneous outcome was PFS (I2 = 69%). After excluding ASTRUM-005 from this study there was no significant heterogeneity (I2 = 0%), which may be because ASTRUM-005 was the most recently conducted trial, and the duration of follow-up was relatively short by the date of data cutoff.

**Figure 10:**
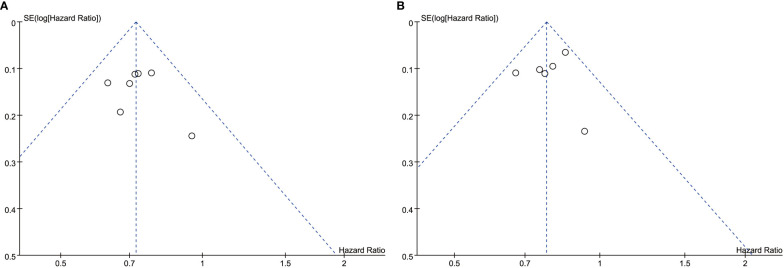
**(A)** Funnel Plot of Overall Survival. **(B)** Funnel Plot of Progression-Free Survival.

The overall quality of the included studies was high. The ones that included RCT were open-label with some risk of bias. Because all trials have been properly randomized, the risk of confounding in RCTs is minimal. Funnel plot asymmetry was not obvious for any outcome (**Figure 10**).

## 4 Discussion

Through genomic analyses of SCLC, investigators have discovered mutations in two tumor suppressor genes in SCLC, p53, and Rb1, which can induce genomic instability and lead to the production of tumor-related antigens (20). Moreover, long-term exposure to smoking carcinogens causes SCLC to become one of the tumors with the highest mutational burden (TMB), which is also closely associated with a large number of potentially immunogenic neoantigens present in the tumors (21, 22). Some researches show an association between high TMB and sensitivity to ICIS, which is associated with the promotion of tumor-specific CD8 + T cell responses (23–25). However, according to the current clinical research results, the correlation between elevated TMB in SCLC and the benefit of ICIs is much lower than that observed in non-small cell lung cancer. This may be due to the limited infiltration of immune cells into the tumor microenvironment, reduced PD-L1 expression, and lack of antigen presentation, which to some extent diminishes the efficacy of immunotherapy in SCLC (26–28). But even so, compared to conventional standard chemotherapy in the past, the application of antibodies that block the pathway of immune checkpoints in SCLC is still promising. Moreover, SCLC is a malignant neuroendocrine tumor with a high frequency of tumor suppressor factors and genetic aberrations of oncogenes, making it feasible to treat SCLC with ICIs.

This study collected 8 RCTs, including CTLA-4 and PD-1/PD-L1 inhibitors, and conducted a meta-analysis of their updated follow-up data (29–31). The results showed that adding ICIs to ES-SCLC first-line chemotherapy significantly improved patients’ OS, PFS, and ORR compared to chemotherapy alone, but patients would experience more AEs. Such results are consistent with the observed application of ICIs in the real world (32). Patients treated with ICIs combined with chemotherapy exhibit higher ORR, indicating that immunotherapy can effectively mobilize the body’s anti-tumor system in ES-SCLC patients, and that can benefit patients more, making ICIs combined with chemotherapy a promising therapeutic strategy for SCLC. Although more grade 3 and above adverse events were observed in patients treated with ICIs plus chemotherapy, after early intervention treatment, most adverse events can also be well resolved. This also reminds us that in clinical applications, more attention should be paid to adverse events during the treatment of ICIs, and patients should be treated as soon as possible and properly managed.

Subgroup analysis showed that ICIs plus chemotherapy significantly improved OS and PFS in patients regardless of age, gender, and performance status. And the PD-1/PD-L1 group performed better than the CTLA-4 group in both efficacy and safety. Results from two studies evaluating CTLA-4 inhibitors demonstrated its ability to prolong PFS, but no clear improvement in OS was observed. This is similar to the results of patients with advanced squamous non-small cell lung cancer receiving Ipilimumab combined chemotherapy. One possible explanation is that Ipilimumab may stimulate early T-cell activation, which may not produce effective antitumor responses in the local tumor environment without corresponding effector T-cell activation. Currently, there are few studies related to CTLA-4 inhibitors, and more studies are needed in the future to determine the role of CTLA-4 inhibitors in the treatment of ES-SCLC.

Of note, our study included the most recent RCTs, such as CAPSTONE-1, which evaluates Adebrelimab, and ASTRUM-005, which evaluates Serplulimab, as well as some recent follow-up results from previous studies that were not contained in previous Meta-analysis studies.

Of course, the included studies and this study also have limitations: there are not many RCTs included; In the included studies, the combined chemotherapy regimen was not completely consistent; The characteristics of eligible patients in each RCT are not the same; Some studies have corporate support, which may lead to unpublished negative results; The impact of potential factors such as smoking history, region, and PD-L1 expression level in patients.

## 5 Conclusion

In conclusion, this study is the latest and most comprehensive study comparing ICIs plus chemotherapy with chemotherapy alone for first-line treatment of ES-SCLC, which verified the efficacy and safety through meta-analysis and provided some drug references for the clinical treatment. However, this study is still flawed and needs to be validated by further large samples and high-quality randomized controlled trials.

## Data availability statement

The raw data supporting the conclusions of this article will be made available by the authors, without undue reservation.

## Author contributions

CC and XF contributed to the conception and design of the study. CC, PT, and JZ collected and assessed the literature. CC and PT performed the statistical analysis. CC wrote the first draft of the manuscript. All authors contributed to the article and approved the submitted version.
